# The impact of global health initiatives on trust in health care provision under extreme resource scarcity: presenting an agenda for debate from a case study of emergency obstetric care in Northern Tanzania

**DOI:** 10.1186/1478-4505-8-14

**Published:** 2010-05-25

**Authors:** Øystein E Olsen

**Affiliations:** 1 Center for International Health, P.O. Box 7024, N-5020, University of Bergen, Norway; 2 Primary Health Care Institute, Iringa, Tanzania for DBL - Center for Health Research and Development, University of Copenhagen, Denmark; 3 Haydom Lutheran Hospital, P.O. Mbulu, Tanzania

## Abstract

**Background:**

Through the nearly three decades that have passed since the Alma Ata conference on Primary Health Care, a wide range of global health initiatives and ideas have been advocated to improve the health of people living in developing countries. The issues raised in the Primary Health Care concept, the Structural Adjustment Programmes and the Health Sector Reforms have all influenced health service delivery. Increasingly however, health systems in developing countries are being described as having collapsed Do the advocated frameworks contribute to this collapse through not adequately including population trust as a determinant of the revival of health services, or are they primarily designed to satisfy the values of other actors within the health care system? This article argues there is an urgent need to challenge common thinking on health care provision under extreme resource scarcity.

**Methods:**

This article sets out to discuss and analyze the described collapse of health services through a brief case study on provision of Emergency Obstetric Care in Northern Tanzania.

**Results:**

The article argues that post the Alma Ata conference on Primary Health Care developments in global health initiatives have not been successful in incorporating population trust into the frameworks, instead focusing narrowly on expert-driven solutions through concepts such as prevention and interventions. The need for quantifiable results has pushed international policy makers and donors towards vertical programmes, intervention approaches, preventive services and quantity as the coverage parameter. Health systems have consequently been pushed away from generalized horizontal care, curative services and quality assurance, all important determinants of trust.

**Conclusions:**

Trust can be restored, and to further this objective a new framework is proposed placing generalized services and individual curative care in the centre of the health sector policy domain. Preventive services are important, but should increasingly be handled by other sectors in a service focused health care system. To facilitate such a shift in focus we should acknowledge that limited resources are available and accept the conflict between population demand and expert opinion, with the aim of providing legitimate, accountable and trustworthy services through fair, deliberative, dynamic and incremental processes. A discussion of the acceptable level of quality, given the available resources, can then be conducted. The article presents for debate that an increased focus on quality and accountability to secure trust is an important precondition for enabling the political commitment to mobilize necessary resources to the health sector.

## Background

A major limiting factor for implementing effective health policies and reforms worldwide is a lack of qualified human resources[1]. Securing human resources for maternal health services is a key component in achieving the Millennium Development Goals (MDG's) by 2015. Specifically, many governments and organizations recognize that human resources have not been targeted sufficiently in previous global development initiatives such as the Sector Wide Approaches and the Poverty Reduction Strategy programmes. It is vital that policy-makers have access to evidence on key aspects of human resource management. However, the best policy choices for health care provision under extreme resource scarcity are not obvious. Results from a previous study of emergency obstetric care in Northern Tanzania [2-4] serve well to illuminate this point. This study aimed at describing the main barriers to implementation of health policy in Tanzania. Provision of qualified health personnel is among the main barriers identified. The study analyzed availability of qualified facilities and human resources necessary for the provision of emergency obstetric care services in six districts. The human resources were categorized according to their ability to conduct a normal delivery (BEmOC) and Caesarean Sections (CEmOC). The results demonstrate, among other things, that there are adequate numbers of qualified staff to provide emergency obstetric care at the aggregated district level compared to Tanzanian human resource policy requirements. However, there are large variations in the availability of qualified staff within these districts and severe understaffing at the dispensary levels in rural districts. The total number of available staff in Tanzania is also very low compared to other African countries.

**Figure 1:**
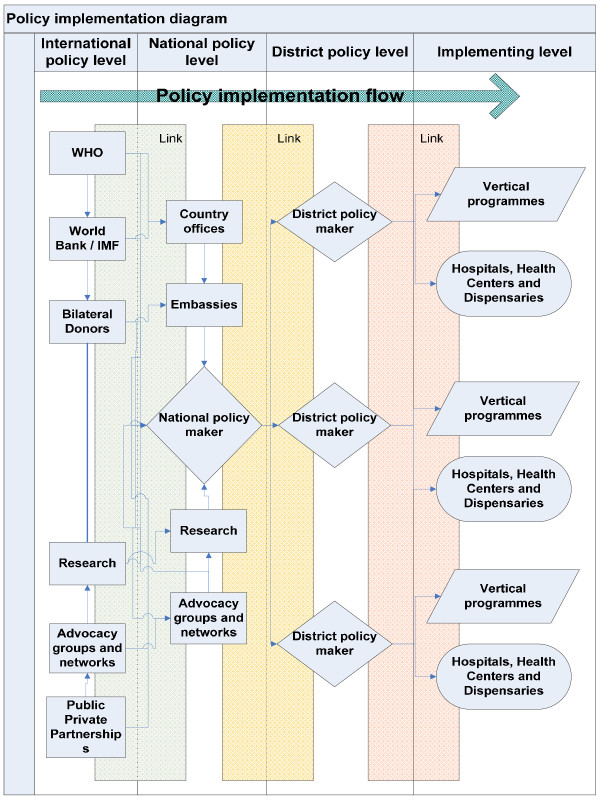
**The policy implementation flow illustrating the main links between the various implementation levels**.

The results must lead us to question the allocation of human resources in this context as well as the leading policy frameworks shaping these allocations. Often the lack of success in policy implementation is ascribed to the barriers caused by the failures and constraints of implementers at lower levels in the health care pyramid [5]. These barriers include low availability of resources, low morale and motivation among staff and low levels of qualifications. Implemented health care is, however, not stronger than the weakest link, and therefore all links need to be scrutinized (see Figure 1). One important link is between policies at national and international levels, where most policies are conceived and defined. What are the consequences of badly conceived, badly promoted and inefficient international policy frameworks? Is it possible that these frameworks are based on values and objectives not synchronized with the other links in the implementation chain, and therefore lack legitimacy and applicability? Given their importance to implementation strategies it is likely that they could represent one of the most important barriers to implementation of effective health care. They therefore need to be subject to challenge and further debate.

Figure 1 is a flow diagram showing the different levels of policy formulation and implementation. The priority setting mechanism for health care delivery in Tanzania is very similar to that of other developing countries. District health facilities, programmes and management systems rely heavily on input from central authorities. Planning directives are widely used, with a substantive top-down, expert based process. In recent years Tanzanian health policy has undergone a reform process in which the districts have enjoyed increased autonomy, but their priority setting flexibility is still very limited. There are ambitions to improve the community voice into this process, but these efforts have only partially been implemented [6,7]. More importantly, however, global health care debates and priority setting frameworks have influenced the Tanzanian priority setting mechanisms at the national level.

Through the nearly three decades that have passed since the Alma Ata conference on Primary Health Care a wide range of global health initiatives and ideas have been advocated to improve the health of people living in developing countries. Primary Health Care has been the leading theoretical concept for delivering health services to vulnerable segments of the population. Later, Structural Adjustment Programmes (SAP) and Health Sector Reforms (HSR) have influenced health service delivery. Increasingly however, health systems in developing countries are being described as having collapsed [5,8-11]. Several questions of importance emerge from these descriptions. What is collapsing, and how is it related to population demand and values? Do the frameworks adequately include population trust or are they designed to satisfy the values of other actors within the health care system? Is it possible to use old frameworks to create change, or do we need to completely re-focus? How can necessary political and individual commitment be mobilized to provide the resources needed for policies to be implemented?

The aims of this paper are twofold:

- to present a case study of the provision of emergency obstetric care in Northern Tanzania and,

- from the lessons learned, to present an agenda for debate about appropriate health policies to reduce implementation barriers for trustworthy and effective health services in situations of extreme scarcity.

## Methods

By presenting the findings as a case study this article hopes to describe the actual present situation in a rural Tanzanian health setting and the possible effects of policy instruments on this situation. The case study methodology is useful in exploring new areas where further research and action are needed. Whereas there exists a rigorous methodological framework for assessing the issues related to EmOC in Tanzania, it is more of a challenge to rigorously apply these findings to what this articles argues to be a major barrier to implementation of effective health care - the policy frameworks themselves. By using the case study methodology it is possible to identify some key issues that, at the very least, are useful for initiating a much needed debate on the role of the policy frameworks. But these issues are also probably useful to explain some of the links between the frameworks and the barriers to implementation. The unit of analysis is the implementation of effective health care to provide emergency obstetric care. Although the study included 129 facilities and six districts, this article only presents one case - the implementation of emergency obstetric care in northern Tanzania. This case nevertheless presents findings from all facilities providing delivery services in the six districts. Emergency Obstetric Care services have been described as a relevant and informative proxy indicator to assess services across the whole health care pyramid, as it encompasses care at both lower and upper levels of the health care system [12-14].

## Results and Discussion

### Case study: the provision of emergency obstetric care in Northern Tanzania

The study used the WHO/UNFPA Guidelines for auditing 129 service providers and to distinguish between basic and comprehensive services [15]. Detailed discussions of material, methods and findings have been published elsewhere [2-4].

**Figure 2:**
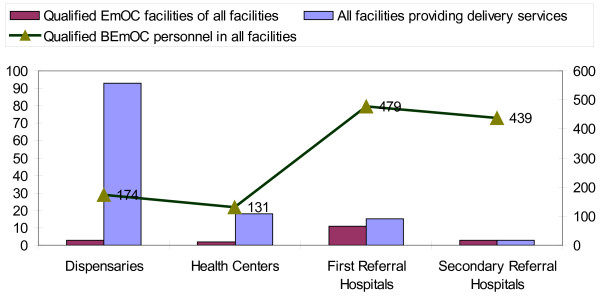
**Qualified EmOC facilities of all facilities in the 6 study districts with distribution of qualified BEmOC personnel**.

The first major observation was that there is an apparently poor mix of services, with a very low availability of basic emergency obstetric care (BEmOC) units and a relatively high availability of comprehensive emergency obstetric care (CEmOC) units. Both services have large urban/rural variation. Only 19 (15%) of 129 facilities are considered to pass the EmOC facility audit of the WHO/UNFPA Guidelines criteria [3]. Disaggregated, these data show that only five of the 111 possible BEmOC facilities (dispensaries and health centres) passed the audit criteria while 14 of the possible 18 CEmOC facilities (first, secondary and tertiary referral hospitals) were qualified. Figure 2 illustrates the availability of qualified health services at facility level. It shows the total available facilities providing delivery services relative to the same facilities qualifying as BEmOC facilities. The figure illustrates the quality gap and potential for improvement at facility level.

The percentage of expected deliveries in EmOC facilities in the study area is 36%, compared with the UN Guidelines minimum accepted threshold of 15%. Most of the facility deliveries were conducted at CEmOC facilities (62%). Only 4% of the facility deliveries were conducted at BEmOC facilities. The rest were conducted at non-qualified EmOC facilities. The distribution of providers shows a much higher availability of qualified facilities in urban districts compared to rural areas, indicating that mothers have to travel long distances to receive adequate services when in need of them[3] Most qualified facilities in rural areas were provided by voluntary agencies, while urban areas were catered to by government agencies.

The second major observation was that nearly 60% of the expected complicated deliveries in the study population were conducted at EmOC qualified health facilities, but only 0.6% of these were conducted at BEmOC facilities. The complicated deliveries were primarily conducted at voluntary agencies facilities. There is an inadequate level of critical services provided, with an average Caesarean Section Rate at 4.6 and only one facility with a Case Fatality Rate below the minimum accepted level of 1.0 [2].

Third, there are adequate human resources allocated for health care provision in Tanzania, if Tanzanian national standards are applied. Compared to similar countries however, Tanzania has a very low availability of health care staff. The presence of qualified human resources in the study area and their distribution are shown (Table 1). Relative to the minimum standards set by Tanzanian authorities[16] the table shows that there are adequate numbers of qualified personnel at health centre levels, but not adequate levels at dispensary levels in rural areas, in which most of the dispensaries are located. This means that most qualified staff are concentrated in a few centralized locations, while those remaining are inequitably and inefficiently distributed in rural areas and in lower-level services. Rural districts have restricted access to government-run health care, mainly because these facilities are understaffed. In addition the dispensaries do not have the managerial capacity to translate the available qualified human resources present into quality activities. The data furthermore show that voluntary agency facilities in these districts tend to have more staff than the government facilities. There is a statistical correlation between availability of qualified human resources and use of services, supporting findings from related research in Tanzania [17-19].

**Table 1:** Distribution of qualified human resources across urban rural districts, health service levels and population.

	Average qualified BEmOC staff per					
	Dispensary	Health Center	First Referral Hospital	Average qualified CEmOC staff per First Referral Hospital	Population per qualified BEmOC staff	Population per qualified CEmOC staff
Urban	4.0	8.9	13.7	1.7	626	7413
Rural	1.6	5.6	39.0	2.6	1949	47416
National standards	2	4				

The table shows the adequacy of qualified BEmOC health personnel at health center levels and above, and the inadequacy of qualified BEmOC health personnel at dispensary level in rural districts. The table also shows large variations in the distribution of qualified staff across the districts.

It is difficult to asses the adequacy of the numbers of qualified human resources at first referral hospital level, but there is reason to suspect that an average number of qualified CEmOC staff of 1.7 in urban districts and 2.6 in rural districts is far too low given the workload at these facilities.

**Figure 3:**
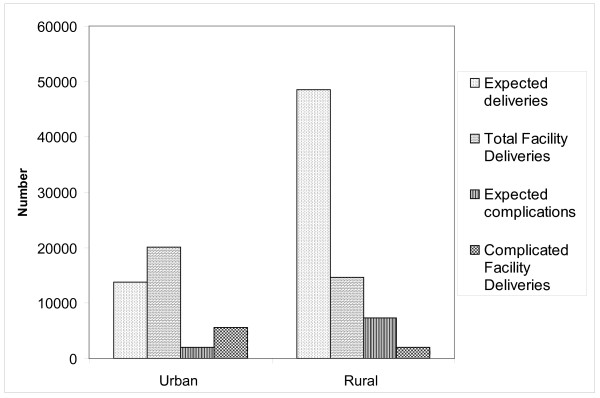
**Actual compared to expected deliveries and complicated deliveries in urban and rural areas**.

Finally the data shows the utilization pattern of the facilities, both across districts and type of facilities. Figure 3 shows that there is a large tendency for pregnant mothers to bypass services provided in rural areas, in order to access services provided in urban areas. This has been shown in previous studies, and is correlated to the mothers' perception of availability of quality services [20].

The data further show that they preferentially choose the qualified facilities in districts where they have a choice between qualified EmOC facilities and non-qualified EmOC facilities.

### Policy implications: an agenda for debate

There is increasing attention being given to the challenges of effective implementation of delivery services [21,22]. This article presents for debate some of the dilemmas faced by developing countries over the past nearly three decades. First, provision of quantity of services rather than quality of services has been the priority, leading to reduced utilization of services, bypassing and serious equity violations. The struggle to be politically correct in international health development circles often leads to infrastructure being placed primarily to satisfy the international donor community's need for distributional indicators. Second, it has been difficult for national and district policy makers to advocate a need for increased funding for curative services, due to the critical attitude of donors towards the high relative proportion of funds already spent for these services. This attitude is coupled with the political strings attached to preventive services. Donors have therefore created global health initiatives incompatible with the severe scarcity of resources and with a disregard for the value of quality curative services. Evidence, for instance, of significant medical benefits from the strong emphasis on antenatal care - without accompanying emergency care - is lacking [23]. Prioritizing preventive and community based health strategies at the expense of curative services and care has arguably led to a lack of trust in, and even near complete contempt for, the same services by the population in question, due to the lack of available services when needed.

As described previously, assessing availability of EmOC is a recognized proxy for describing the health services in general, or horizontal services. Thirdly therefore, it can be argued that in the study area there is almost a collapse of horizontal services, while there exists a plethora of well-funded vertical programmes and single interventions. Sometimes there are even more than one vertical programme per disease (e.g. Malaria and HIV/AIDS).

Finally, and most importantly, the case study also illustrates the priority setting dilemma of local health managers. Although donor funded infrastructure aims at providing a broad referral base, there has not been an adequate supply of qualified human resources to man these facilities. Policy makers in Tanzania have therefore made the only obvious decision possible, namely to put qualified people where they can reach the most people with adequate equipment - i.e. in hospitals in central urban locations.

#### Trust as a platform for debate

Social institutions and values, including trust, are increasingly being given attention when describing health policy [24-29]. Trust is one of the ultimate tests of success or failure of a given health system. Many commentators on trust will argue that what is actually important is the notion of trustworthiness [30]. Harding describes the many notions of trust and very importantly defines it as a cognitive rather than a primitive concept. To say that it is not primitive means that it can and must be reduced to other notions to adequately be described. To say that it is cognitive means that trusting in something ultimately depends on our knowledge about it, and particularly knowledge about why it should be trustworthy.

The importance of trust being cognitive is also that we cannot actively choose to trust, as our level of knowledge defines our level of trust or distrust. Harding continues to reduce the elements of trust and trustworthiness into some very crucial concepts. These are the concepts of reliance, risk taking, expectation and confidence. All of these elements are measurable and of critical importance to e.g. health care and government. Harding also underlines the importance of accepting distrust as a measure of trustworthiness, particularly in the relationship between the individual and government. He argues that, while it is not possible to fully understand and have adequate knowledge of why a government should be trusted, it is relatively easy to have knowledge of why a government should be distrusted. It is therefore easier to invoke distrust than trust.

Trust, or trustworthiness, is a function of the level of responsiveness of the health system to the population's needs and demands. Adequate delivery of quality services and acceptable access to these prioritized services are key determinants of responsiveness [31]. Trust is necessary not only for its own sake, but also to improve quality and efficiency of health care [32]. Trust, as summarized by Lucy Gilson, is crucial to health service delivery in that it contributes to improved co-operation within the health system and between the actors in delivering services [27]. Trust-based health services also enable development on a broader scale within communities. Trust is based on the values creating the social fabric of a society, including peace, security, justice and health [33]. Population health is based not only on the sum of individual's health status and health risks, but also on the sum of the contribution to population health of values such as trust. Presence, or absence of, values are in themselves determinants of population health [24,34-36].

As a value domain, trust is not easily defined or quantified. Neither are the determinants of trust easily identified. Common use of the term is closely related to concepts such as confidence, reliance and risk [37]. There are, however, two elements of trust that can usefully be studied and improved in practically all health policy formulations. Harding roughly divides these into:

- elements of trust that provide explanations of behaviour and social institutions and

- the concept of trust as the outcome of behaviours guided by a motivational factor or a sense of morality that is important in its own right.

This last point is particularly important as it means that each actor may have an inherent interest in being trusted, either because it is part of their sense of morality or because it creates an outcome to their benefit. Giddens defines two similar notions as "faceless" trust and "facework" trust [29]. "Faceless" describes the fabric of society and systems (including health systems) while "facework" describes the interpersonal dimensions between people (doctor and patient). "Faceless" is concerned with knowledge (or ignorance) and what Giddens terms "backstage expertise", determining in this case the health services system and its trustworthiness. "Facework" is concerned with the "access points" at which the delivery of the system is presented. The concepts involved here are the experience, attitude and expertise of the provider as well as the level of focused interaction between the provider and the user.

For the purpose of this debate the determinants need to reflect these main dimensions of trust. Important determinants will include quality of services and access to these services, the legitimacy of the services and the priority setting mechanisms defining the objectives and use of resources towards these objectives. Most of the empirical work presented previously in the article is concerned with describing implementation and utilization of services as a proxy for, or an expression of, trust.

Although one should always be careful in generalizing from a single case study, experience from elsewhere and the results presented in this article allow questioning of the underlying premises of the priority setting mechanism determining the distribution of qualified human resources. Five issues are presented as an agenda for debate, deliberately designed to challenge common thinking in this area.

##### 1. Restoring trust through legitimate decision-making

Health systems in many developing countries are falling apart. As described earlier in the article, one of the most crucial issues at stake is trust. A re-orientation of policies and practices are needed to restore trust and secure better services for the poor. As a first point for debate, old global health initiatives post Alma Ata are compared and contrasted with the more recent developments in health policy to identify new mechanisms for restoring trust in health care (Table 2).

**Table 2:** Post Alma Ata developments versus new mechanisms for securing trust in health care in developing countries.

Post Alma Ata developments	Mechanisms for securing trust
**Interventions and Services**	
Post Alma Ata narrow focus on interventions by researchers and policy makers leaves services non-prioritized	Priority to services rather than interventions Better tools for monitoring and evaluation of generalized services
**Vertical and Horizontal**	

Priority to vertical programmes has contributed to a collapse of horizontal services	Includes horizontal services
Vertical programmes easily deteriorate existing health services and create large transaction costs at higher levels	Aims at synergy between essential vertical programmes and generalized horizontal services
	
**Prevention and Cure**	

A policy focus primarily on prevention has led to a deterioration of curative services	Higher priority to clinical curative services
Prevention interventions often health expert driven Curative services often excluded through funding mechanisms	Maintains focus on citizens and implementers opinions Accepts gap and seeks compromise between experts opinion and patient demand through reasonable, deliberative processes
	Maintains relevant preventive activities, with higher emphasis on their relevance to other sectors
**Quality and Quantity**	

Frameworks have almost focused on quantity and coverage before quality	Higher priority to quality assurance mechanisms
Isolated focus on quantity is not pro-poor	Securing quality before increasing coverage
Vertical programmes easily funded and researched due to easily identifiable objectives and quantifiable results	Maintains a dynamic and incremental focus aimed at describing complex structures and continuous improvement
	
**Priority setting mechanism**	

PHC based on social justice principles without adequate focus on availability of resources and tools to ensure implementation and social support	Priority setting in response to available resources Aims at fair and efficient priority setting Recognizes the importance of legitimacy and public support
Providers not accountable to patients and the public	Aims at increasing accountability to all affected parties through deliberative processes and transparent decision making
	

A shift in priorities is needed, and this article suggests procedural mechanisms for making the required changes. Although it is beyond the scope of this article to describe how procedures for legitimate priority setting could be implemented, it should be mentioned that the formative research on such frameworks has moved on to provide practical tools for district health planners [38,39]. Their importance has been demonstrated both in developing and developed countries [38,40-42]. They are currently being tested in the Tanzanian district health priority setting context, among others [43,44].

##### 2. Higher priority to health care services, not interventions

**Figure 4:**
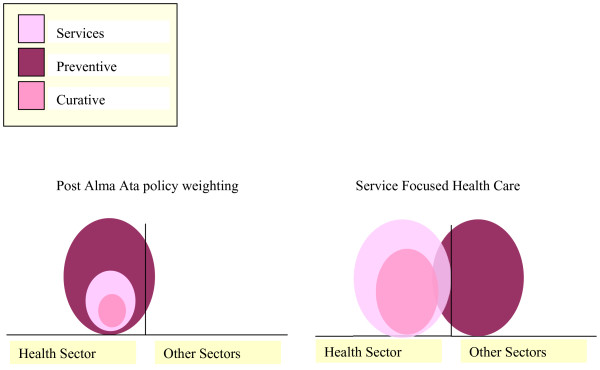
**Changes to policy weight now given to services, preventive interventions and curative interventions during the Primary Health Care and post Primary Health Care eras in contrast to the policy weight proposed by a Service Focused Health Care framework**.

The shift in focus to services as the prime objective of health policy is illustrated in Figure 4. The figure shows policy weight given to services and interventions by post Alma Ata (primary health care, market oriented and interventionist approaches) de facto policies (i.e. not the primary intentions but the actual policy weight given). Neither the market-oriented nor the interventionist approaches have markedly differed in their policy focus. The figure further shows the shift in policy attention proposed by a framework focusing on trust.

The figure further illustrates the need for re-orienting priorities within the health sector to services and individual curative care. Through this approach there is a potential for both releasing more resources to the health sector and increasing trust in health services, thereby contributing efficacy gains to the system from both the supplier and customer. Although not always recognized, giving higher priority to generalized services remains an underlying premise for good interventions. The lack of tools and methodology to adequately describe, monitor and evaluate horizontal coverage has also influenced researchers to embrace the intervention concept rather than broader services. It is more convenient to study clinical efficacy and cost-effectiveness of a limited set of interventions than to study a complete service composite[45].

##### 3. Higher priority to the provision of visible and tangible clinical curative services

One mechanism for securing trust in healthcare services would be a lower policy emphasis on preventive services within the health care sector - not because they are unimportant, but because they should increasingly be handled in other sectors where they belong. Although health care is also important to economic growth [46], the most important determinants of health are found outside the health sector [47-50]. Governments need to fully embrace the fact that other sectors more effectively could take responsibility for the health of their population. Preventive programmes should not be downplayed, but rather "outsourced" to other sectors. Examples of this could include the distribution of bed nets in Malaria control, information and education activities in HIV/AIDS control, improving education and access to services in maternal mortality control, sanitation and hygiene activities and injury prevention, to mention only a few. Higher priority to curative care can induce increased motivation to contribute to population health in other sectors. Using highly qualified health personnel to coordinate preventive activities may be inefficient. Meagre qualified resources should instead be used where they matter most to the supply of individually oriented clinical services. Let qualified health personnel do what they are trained to do - cure and care. Preventive activities needing medical expertise should, however, still be handled by the health sector. The World Development Report of 2004 [1] and the Health Performance Assessment framework [31] go far, but not far enough, in emphasising this point. There is increasing recognition that adequate treatment, once ill, is of utmost importance in adequately providing care for the poor[51]. Although not the intention of Alma Ata, the impression has nevertheless been maintained that Primary Health Care is mainly associated with low-level non-curative and therefore cost effective care. Hospital services have been considered less cost effective or ignored and often excluded in the health service debate[52]. Consequently they have been abandoned by most stakeholders. It has been successfully shown however, that both preventive and curative services could be cost effective and cost ineffective[53,54].

##### 4. Higher priority to the demand of citizen's, not the policy maker's benefit

It is important to ask which actors the present global health initiatives seem to benefit. Preventive services and cost-effective vertical intervention programs are arguably mostly satisfying the needs of the major donors and policy makers. Focusing on higher returns of invested resources on a greater number of the population is gratifying to policy makers remote from the individual suffering from a disease or condition not prioritised through these processes. Paradoxically, a focus on general services and the individual generalized curative services is an important determinant of population trust, while at the same time a politically incorrect perspective to embrace in international health policy and donor environments.

##### 5. Higher priority to quality assurance mechanisms not quantity assurance mechanisms

A promising mechanism for securing trust is the quality assurance methodology, in which indicators and tools are sought to adequately provide proxies for the evaluation and improvement of health services, rather than interventions alone [55-58]. Quality assurance tools maintain a dynamic and incremental focus aimed at describing complex structures and continuous improvement. Although growing, there is unfortunately little research on the tools required to measure improvements of care in developing countries[45].

Moreover, quality should be secured before quantity of services. Given the low number of qualified health personnel, such as doctors, available in a given developing country, where do we place them? This article argues that they primarily should be placed at the lowest possible level in the health care pyramid where adequate facilities are available to make full use of their skills. In other words, they should provide quality services in facilities adequately equipped to facilitate such services. The level of services, and subsequently positioning of cadres at each facility level, will be a result of the minimum acceptable level of quality and available resources.

Our case study demonstrates that resources could be moved up the pyramid from dispensaries to health centres in order to increase availability of quality services [4]. It further shows that to meet safe obstetric delivery objectives a minimum of BEmOC qualified personnel must be available in the facility. If delivery services are to be provided at the base of the pyramid, then facilities without qualified BEmOC personnel cannot be accepted. Only as available resources increase can they be placed lower down in the health care pyramid. Under extreme resource scarcity, one cannot rely solely on standard primary care strategies. A low level of available resources implies an increased attention to the delivery of quality services at higher levels of the health care pyramid. The present focus on channelling meagre resources to lower levels of the health care pyramid is ineffective, albeit though the visions and objectives of providing accessible services to the poor are commendable. Low quality services erode trust. Services of inadequate quality are not "pro-poor" even if they are accessible to all. Demand is responsive to quality of care,[59,60] and poor quality will eventually lead to bypassing of low-quality facilities to higher-quality facilities, and thus also increase inequity of access[20] and the effect of health systems on iatrogenic poverty[61].

## Conclusions

This article sets out an agenda for debate on the role of trust as a determinant of the revival of health services in a context of severe resource constraints. It argues that global health initiatives over the past three decades since Alma Ata do not adequately address trust as a determinant, and therefore contribute to the collapse of health services in developing countries. There is a need for further research and debate on the role of trust in improving health services, as shown in the case study from Tanzania and the discussion in this article. How can health policies secure trust, and with it improve access to generalized quality curative services and care? These health care domains are in immediate danger of collapsing, with the consequent further collapse of population trust. Attention should be given to increasing the availability of resources, but also to how the resources available are prioritized to suit the needs and expectations of the public. To further facilitate a focus on trust we need to acknowledge limited availability of resources and address the conflict between people's demand and expert opinion.

## Competing interests

The author declares that they have no competing interests.
